# Tris(4-methyl­phen­yl)phosphine selenide

**DOI:** 10.1107/S1600536810050567

**Published:** 2010-12-08

**Authors:** Alfred Muller

**Affiliations:** a Research Centre in Synthesis and Catalysis, Department of Chemistry, University of Johannesburg (APK Campus), PO Box 524, Auckland Park, Johannesburg 2006, South Africa

## Abstract

In the title mol­ecule, C_21_H_21_PSe or PSe(C_7_H_7_)_3_, the P atom has a distorted PSeC_3_ tetra­hedral environment, formed by the Se atom [P=Se = 2.1119 (5) Å] and three aryl rings. Two short intra­molecular C—H⋯Se contacts occur. In the crystal, weak inter­molecular C—H⋯Se inter­actions link the mol­ecules into zigzag double chains propagating in [100]. The previous report of this structure [Zhdanov *et al.* (1953 ▶). *Dokl. Akad. Nauk SSSR* (*Russ*.) (*Proc. Nat. Acad. Sci. USSR*), **92**, 983–985] contained no geometrical data.

## Related literature

For the previous structure determination, see: Zhdanov *et al.* (1953 ▶). For background to phospho­rus- and selenium-containing ligands, see: Muller *et al.* (2006 ▶, 2008 ▶); Roodt *et al.* (2003 ▶). For a description of the Cambridge Structural Database, see: Allen (2002 ▶); For ligand cone angles, see: Tolman (1977 ▶).
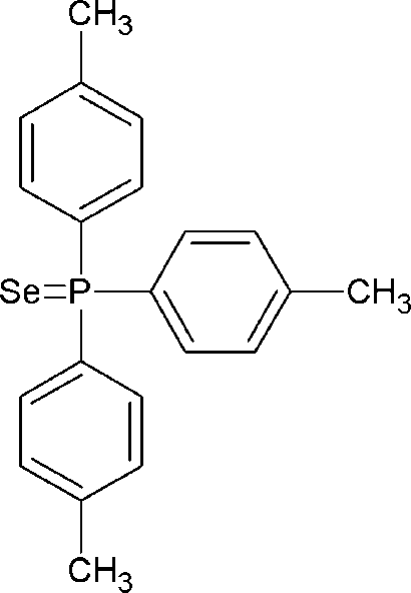


## Experimental

### 

#### Crystal data

C_21_H_21_PSe
*M* *_r_* = 383.31
Monoclinic, 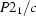

*a* = 9.8330 (4) Å
*b* = 19.0584 (9) Å
*c* = 11.9136 (4) Å
β = 124.969 (2)°
*V* = 1829.55 (13) Å^3^
*Z* = 4
Mo *K*α radiation
μ = 2.14 mm^−1^
*T* = 100 K
0.36 × 0.14 × 0.13 mm

#### Data collection

Bruker X8 APEXII 4K KappaCCD diffractometer
Absorption correction: multi-scan (*SADABS*; Bruker, 2004 ▶) *T*_min_ = 0.513, *T*_max_ = 0.769
12931 measured reflections
4555 independent reflections
3748 reflections with *I* > 2σ(*I*)
*R*_int_ = 0.032

#### Refinement

*R*[*F* ^2^ > 2σ(*F* ^2^)] = 0.031
*wR*(*F* ^2^) = 0.073
*S* = 1.03
4555 reflections
211 parameters
H-atom parameters constrained
Δρ_max_ = 0.46 e Å^−3^
Δρ_min_ = −0.30 e Å^−3^

### 

Data collection: *APEX2* (Bruker, 2005 ▶); cell refinement: *SAINT-Plus* (Bruker, 2004 ▶); data reduction: *SAINT-Plus* and *XPREP* (Bruker 2004 ▶); program(s) used to solve structure: *SIR97* (Altomare *et al.*, 1999 ▶); program(s) used to refine structure: *SHELXL97* (Sheldrick, 2008 ▶); molecular graphics: *DIAMOND* (Brandenburg & Putz, 2005 ▶); software used to prepare material for publication: *WinGX* (Farrugia, 1999 ▶).

## Supplementary Material

Crystal structure: contains datablocks global, I. DOI: 10.1107/S1600536810050567/hb5761sup1.cif

Structure factors: contains datablocks I. DOI: 10.1107/S1600536810050567/hb5761Isup2.hkl

Additional supplementary materials:  crystallographic information; 3D view; checkCIF report

## Figures and Tables

**Table 1:** Hydrogen-bond geometry (Å, °)

*D*—H⋯*A*	*D*—H	H⋯*A*	*D*⋯*A*	*D*—H⋯*A*
C12—H12⋯Se	0.95	3.04	3.495 (2)	111
C12—H12⋯Se^i^	0.95	3.18	3.890 (2)	133
C2—H2*B*⋯Se^ii^	0.98	3.09	4.067 (2)	176
C36—H36⋯Se	0.95	3.13	3.556 (2)	109

Symmetry codes: (i) 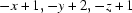; (ii) 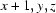.

**Fig. 1.**
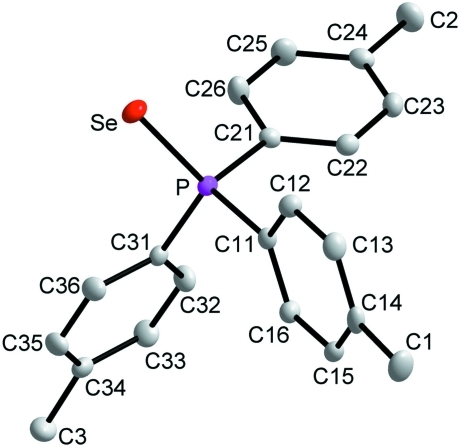
View of (I) (50% probability displacement ellipsoids). H atoms have been omitted for clarity. For the C atoms, the first digit indicates the ring number and the second digit indicates the position of the atom in the ring.

**Fig. 2.**
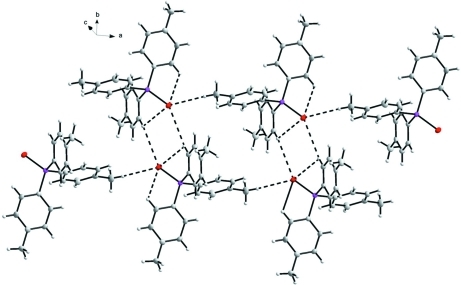
Packing diagram of (I) showing the dimeric units formed and the propagation along [100] with H···Se links as dashed lines.

## supplementary crystallographic information

### Comment

There has been extensive development in understanding the transition metal phosphorous bond by various groups, including our own, with various techniques such as single-crystal X-ray crystallography, multi nuclear NMR and IR (Roodt *et al.*, 2003). As part of this systematic investigation we are now also studying selenium bonded phosphorus ligands (see Muller *et al.* 2008) This way there is no steric crowding effect, abeit crystal packing effects, as normally found in transition metal complexes with bulky ligands, *e.g.* in *trans*-[Rh(CO)Cl{P(OC_6_H_5_)_3_}_2_] coneangles variation from 156° to 167° was observed for the two phosphite ligands (Muller, *et al.* 2006). The *J*(^31^P-^77^Se) coupling can also be used as an additional probe to obtain more information regarding the nature of the phosphorous bond. Reported here, as part of the above continuing study, the single-crystal structure of the compound P(4—Me—C_6_H_3_)_3_ is presented. This was done as no geometrical data are available from the CCDC (Cambridge Structural Database; Version 5.31, update of August; Allen, 2002) on the previously published structure reported by Zhdanov *et al.*, 1953.

Crystals of the title compound, (I), packs in the *P*2_1_/*c* (*Z* = 4) space group with the molecules lying on general positions. All geometrical features of the molecule (Allen, 2002) are as expected with the selenium atom and the three aryl groups adopting a distorted arrangement about phosphorous (see Fig. 1 and Table 1). The cone angle was found to be 161.1° when the Se—P distance is adjusted to 2.28 Å (the default value used in Tolman, 1977).

The packing in the unit cell show Se-atoms forming dimeric units with bi-furcated H-atoms of C12. These units are propagated along the [100] direction with additional weak C—H···Se interactions (See Table 2, Fig. 2).

### Experimental

SeP(4-Me-C_6_H_3_)_3_ and KSeCN were purchased from Sigma-Aldrich and used without purification. Eqimolar amounts of KSeCN and the SeP(4-Me—C_6_H_3_)_3_ compound (*ca* 0.04 mmol) were dissolved in the minimum amounts of methanol (10 – 20 ml). The KSeCN solution was added drop wise (5 min.) to the phosphine solution with stirring at room temperature. The final solution was left to evaporate slowly until dry to give colourless blocks.

Analytical data: ^31^P {H} NMR (CDCl_3_, 121.42 MHz):δ = 34.60 (t, ^1^J_P—Se_ = 717.6 Hz)

### Refinement

The aromatic and methylene H atoms were placed in geometrically idealized positions (C—H = 0.93 – 0.98 Å) and constrained to ride on their parent atoms with *U*_iso_(H) = 1.2*U*_eq_(C) and *U*_iso_(H) = 1.5*U*_eq_(C) respectively, with torsion angles refined from the electron density for the methyl groups. The highest residual electron density is located 0.94 Å from Se.

### Crystal data

**Table:**

C_21_H_21_PSe	*F*(000) = 784
*M**_r_* = 383.31	*D*_x_ = 1.392 Mg m^−^^3^
Monoclinic, *P*2_1_/*c*	Mo *K*α radiation, λ = 0.71073 Å
Hall symbol: -P 2ybc	Cell parameters from 3903 reflections
*a* = 9.8330 (4) Å	θ = 2.5–28.3°
*b* = 19.0584 (9) Å	µ = 2.14 mm^−^^1^
*c* = 11.9136 (4) Å	*T* = 100 K
β = 124.969 (2)°	Block, colorless
*V* = 1829.55 (13) Å^3^	0.36 × 0.14 × 0.13 mm
*Z* = 4	

### Data collection

**Table:**

Bruker X8 APEXII 4K KappaCCD diffractometer	4555 independent reflections
graphite	3748 reflections with *I* > 2σ(*I*)
Detector resolution: 8.4 pixels mm^-1^	*R*_int_ = 0.032
ω and φ scans	θ_max_ = 28.4°, θ_min_ = 2.1°
Absorption correction: multi-scan (*SADABS*; Bruker, 2004)	*h* = −13→9
*T*_min_ = 0.513, *T*_max_ = 0.769	*k* = −25→24
12931 measured reflections	*l* = −15→15

### Refinement

**Table:**

Refinement on *F*^2^	Primary atom site location: structure-invariant direct methods
Least-squares matrix: full	Secondary atom site location: difference Fourier map
*R*[*F*^2^ > 2σ(*F*^2^)] = 0.031	Hydrogen site location: inferred from neighbouring sites
*wR*(*F*^2^) = 0.073	H-atom parameters constrained
*S* = 1.03	*w* = 1/[σ^2^(*F*_o_^2^) + (0.0326*P*)^2^ + 0.6745*P*] where *P* = (*F*_o_^2^ + 2*F*_c_^2^)/3
4555 reflections	(Δ/σ)_max_ = 0.006
211 parameters	Δρ_max_ = 0.46 e Å^−^^3^
0 restraints	Δρ_min_ = −0.30 e Å^−^^3^

### Special details

**Table:**

Experimental. The intensity data was collected on a Bruker X8 Apex II 4 K Kappa CCD diffractometer using an exposure time of 10 s/frame. A total of 640 frames were collected with a frame width of 0.5° covering up to θ = 28.41° with 99.1% completeness accomplished.
Geometry. All e.s.d.'s (except the e.s.d. in the dihedral angle between two l.s. planes) are estimated using the full covariance matrix. The cell e.s.d.'s are taken into account individually in the estimation of e.s.d.'s in distances, angles and torsion angles; correlations between e.s.d.'s in cell parameters are only used when they are defined by crystal symmetry. An approximate (isotropic) treatment of cell e.s.d.'s is used for estimating e.s.d.'s involving l.s. planes.
Refinement. Refinement of *F*^2^ against ALL reflections. The weighted *R*-factor *wR* and goodness of fit *S* are based on *F*^2^, conventional *R*-factors *R* are based on *F*, with *F* set to zero for negative *F*^2^. The threshold expression of *F*^2^ > σ(*F*^2^) is used only for calculating *R*-factors(gt) *etc*. and is not relevant to the choice of reflections for refinement. *R*-factors based on *F*^2^ are statistically about twice as large as those based on *F*, and *R*- factors based on ALL data will be even larger.

### Fractional atomic coordinates and isotropic or equivalent isotropic displacement parameters (Å^2^)

**Table:**

	*x*	*y*	*z*	*U*_iso_*/*U*_eq_	
Se	0.35082 (2)	0.927791 (11)	0.29390 (2)	0.01903 (7)	
P	0.53055 (6)	0.85475 (3)	0.32421 (5)	0.01317 (11)	
C1	1.0689 (3)	0.81013 (13)	0.9344 (2)	0.0271 (5)	
H1A	1.1148	0.8552	0.9806	0.041*	
H1B	1.1584	0.7806	0.9472	0.041*	
H1C	1.0164	0.7866	0.9735	0.041*	
C2	0.8493 (3)	0.94613 (13)	0.0318 (2)	0.0278 (5)	
H2A	0.8035	0.9171	−0.0502	0.042*	
H2B	0.9700	0.9400	0.0919	0.042*	
H2C	0.8233	0.9955	0.0050	0.042*	
C3	0.2520 (3)	0.55993 (11)	0.1189 (2)	0.0255 (5)	
H3A	0.2297	0.5513	0.0286	0.038*	
H3B	0.1492	0.5544	0.1132	0.038*	
H3C	0.3348	0.5263	0.1850	0.038*	
C11	0.6948 (2)	0.84178 (10)	0.50352 (18)	0.0138 (4)	
C12	0.7425 (2)	0.89697 (11)	0.59514 (19)	0.0172 (4)	
H12	0.6909	0.9415	0.5629	0.021*	
C13	0.8654 (2)	0.88729 (11)	0.73382 (19)	0.0193 (4)	
H13	0.8978	0.9255	0.7953	0.023*	
C14	0.9415 (2)	0.82244 (12)	0.78349 (19)	0.0182 (4)	
C15	0.8954 (2)	0.76788 (11)	0.6907 (2)	0.0184 (4)	
H15	0.9489	0.7237	0.7228	0.022*	
C16	0.7729 (2)	0.77686 (11)	0.55230 (19)	0.0169 (4)	
H16	0.7421	0.7388	0.4907	0.020*	
C21	0.6314 (2)	0.88090 (10)	0.24281 (19)	0.0147 (4)	
C22	0.8020 (2)	0.88731 (10)	0.31340 (19)	0.0169 (4)	
H22	0.8714	0.8766	0.4084	0.020*	
C23	0.8724 (3)	0.90939 (11)	0.2458 (2)	0.0192 (4)	
H23	0.9895	0.9143	0.2957	0.023*	
C24	0.7742 (3)	0.92422 (10)	0.1068 (2)	0.0181 (4)	
C25	0.6036 (3)	0.91762 (12)	0.0366 (2)	0.0245 (5)	
H25	0.5346	0.9275	−0.0587	0.029*	
C26	0.5322 (3)	0.89686 (12)	0.1030 (2)	0.0246 (5)	
H26	0.4149	0.8934	0.0533	0.030*	
C31	0.4463 (2)	0.76849 (10)	0.25790 (18)	0.0138 (4)	
C32	0.4990 (3)	0.72710 (11)	0.1928 (2)	0.0191 (4)	
H32	0.5801	0.7446	0.1806	0.023*	
C33	0.4336 (3)	0.66068 (11)	0.1462 (2)	0.0210 (4)	
H33	0.4693	0.6334	0.1009	0.025*	
C34	0.3169 (2)	0.63330 (11)	0.16437 (19)	0.0177 (4)	
C35	0.2645 (3)	0.67488 (11)	0.2292 (2)	0.0203 (4)	
H35	0.1842	0.6570	0.2420	0.024*	
C36	0.3273 (2)	0.74153 (11)	0.2750 (2)	0.0187 (4)	
H36	0.2894	0.7691	0.3183	0.022*	

### Atomic displacement parameters (Å^2^)

**Table:**

	*U*^11^	*U*^22^	*U*^33^	*U*^12^	*U*^13^	*U*^23^
Se	0.01853 (11)	0.01859 (11)	0.01791 (10)	0.00640 (8)	0.00924 (9)	0.00104 (8)
P	0.0130 (2)	0.0139 (2)	0.0114 (2)	0.00109 (19)	0.0064 (2)	0.00008 (17)
C1	0.0196 (11)	0.0416 (14)	0.0134 (10)	0.0004 (10)	0.0055 (9)	0.0040 (9)
C2	0.0286 (12)	0.0355 (14)	0.0263 (11)	−0.0021 (10)	0.0198 (10)	0.0042 (9)
C3	0.0248 (11)	0.0173 (11)	0.0307 (12)	−0.0015 (9)	0.0137 (10)	−0.0017 (8)
C11	0.0125 (9)	0.0181 (10)	0.0111 (8)	−0.0012 (7)	0.0070 (7)	0.0004 (7)
C12	0.0183 (10)	0.0191 (10)	0.0160 (9)	0.0017 (8)	0.0109 (8)	0.0010 (7)
C13	0.0193 (10)	0.0255 (11)	0.0139 (9)	−0.0025 (9)	0.0100 (9)	−0.0049 (8)
C14	0.0118 (9)	0.0299 (12)	0.0132 (9)	−0.0026 (8)	0.0073 (8)	0.0014 (8)
C15	0.0132 (9)	0.0198 (10)	0.0192 (10)	0.0014 (8)	0.0076 (8)	0.0062 (8)
C16	0.0146 (9)	0.0177 (10)	0.0162 (9)	−0.0003 (8)	0.0076 (8)	−0.0004 (7)
C21	0.0173 (10)	0.0140 (9)	0.0135 (9)	−0.0011 (8)	0.0091 (8)	−0.0005 (7)
C22	0.0177 (10)	0.0171 (10)	0.0142 (9)	0.0002 (8)	0.0082 (8)	0.0002 (7)
C23	0.0151 (10)	0.0212 (11)	0.0208 (10)	−0.0004 (8)	0.0100 (9)	−0.0001 (8)
C24	0.0237 (10)	0.0150 (10)	0.0195 (10)	−0.0008 (8)	0.0147 (9)	0.0002 (7)
C25	0.0232 (11)	0.0330 (13)	0.0143 (9)	−0.0013 (10)	0.0091 (9)	0.0047 (8)
C26	0.0166 (10)	0.0350 (13)	0.0175 (10)	−0.0041 (9)	0.0070 (9)	0.0041 (9)
C31	0.0114 (9)	0.0154 (10)	0.0117 (8)	0.0009 (7)	0.0050 (8)	0.0003 (7)
C32	0.0189 (10)	0.0213 (11)	0.0227 (10)	−0.0017 (8)	0.0152 (9)	−0.0038 (8)
C33	0.0230 (11)	0.0214 (11)	0.0236 (10)	0.0005 (9)	0.0163 (9)	−0.0051 (8)
C34	0.0145 (9)	0.0165 (10)	0.0163 (9)	0.0021 (8)	0.0054 (8)	0.0023 (7)
C35	0.0212 (10)	0.0192 (11)	0.0273 (11)	−0.0004 (9)	0.0179 (10)	0.0022 (8)
C36	0.0213 (11)	0.0198 (10)	0.0216 (10)	0.0013 (8)	0.0161 (9)	0.0001 (8)

### Geometric parameters (Å, °)

**Table:**

Se—P	2.1119 (5)	C15—C16	1.387 (3)
P—C31	1.806 (2)	C15—H15	0.9500
P—C21	1.810 (2)	C16—H16	0.9500
P—C11	1.8106 (19)	C21—C22	1.385 (3)
C1—C14	1.509 (3)	C21—C26	1.398 (3)
C1—H1A	0.9800	C22—C23	1.395 (3)
C1—H1B	0.9800	C22—H22	0.9500
C1—H1C	0.9800	C23—C24	1.386 (3)
C2—C24	1.509 (3)	C23—H23	0.9500
C2—H2A	0.9800	C24—C25	1.386 (3)
C2—H2B	0.9800	C25—C26	1.383 (3)
C2—H2C	0.9800	C25—H25	0.9500
C3—C34	1.503 (3)	C26—H26	0.9500
C3—H3A	0.9800	C31—C36	1.395 (3)
C3—H3B	0.9800	C31—C32	1.397 (3)
C3—H3C	0.9800	C32—C33	1.384 (3)
C11—C12	1.390 (3)	C32—H32	0.9500
C11—C16	1.395 (3)	C33—C34	1.386 (3)
C12—C13	1.391 (3)	C33—H33	0.9500
C12—H12	0.9500	C34—C35	1.394 (3)
C13—C14	1.389 (3)	C35—C36	1.381 (3)
C13—H13	0.9500	C35—H35	0.9500
C14—C15	1.391 (3)	C36—H36	0.9500
			
C31—P—C21	105.74 (9)	C15—C16—C11	120.03 (18)
C31—P—C11	105.50 (9)	C15—C16—H16	120.0
C21—P—C11	106.11 (9)	C11—C16—H16	120.0
C31—P—Se	113.32 (6)	C22—C21—C26	118.64 (18)
C21—P—Se	112.88 (7)	C22—C21—P	122.94 (14)
C11—P—Se	112.64 (7)	C26—C21—P	118.40 (15)
C14—C1—H1A	109.5	C21—C22—C23	120.34 (18)
C14—C1—H1B	109.5	C21—C22—H22	119.8
H1A—C1—H1B	109.5	C23—C22—H22	119.8
C14—C1—H1C	109.5	C24—C23—C22	120.98 (19)
H1A—C1—H1C	109.5	C24—C23—H23	119.5
H1B—C1—H1C	109.5	C22—C23—H23	119.5
C24—C2—H2A	109.5	C25—C24—C23	118.43 (19)
C24—C2—H2B	109.5	C25—C24—C2	120.15 (18)
H2A—C2—H2B	109.5	C23—C24—C2	121.41 (19)
C24—C2—H2C	109.5	C26—C25—C24	121.11 (19)
H2A—C2—H2C	109.5	C26—C25—H25	119.4
H2B—C2—H2C	109.5	C24—C25—H25	119.4
C34—C3—H3A	109.5	C25—C26—C21	120.48 (19)
C34—C3—H3B	109.5	C25—C26—H26	119.8
H3A—C3—H3B	109.5	C21—C26—H26	119.8
C34—C3—H3C	109.5	C36—C31—C32	118.80 (18)
H3A—C3—H3C	109.5	C36—C31—P	118.96 (15)
H3B—C3—H3C	109.5	C32—C31—P	122.22 (15)
C12—C11—C16	119.12 (17)	C33—C32—C31	120.29 (19)
C12—C11—P	119.65 (15)	C33—C32—H32	119.9
C16—C11—P	121.22 (14)	C31—C32—H32	119.9
C11—C12—C13	120.32 (19)	C32—C33—C34	121.15 (19)
C11—C12—H12	119.8	C32—C33—H33	119.4
C13—C12—H12	119.8	C34—C33—H33	119.4
C14—C13—C12	120.87 (19)	C33—C34—C35	118.29 (19)
C14—C13—H13	119.6	C33—C34—C3	120.78 (19)
C12—C13—H13	119.6	C35—C34—C3	120.90 (19)
C13—C14—C15	118.43 (18)	C36—C35—C34	121.24 (19)
C13—C14—C1	121.52 (19)	C36—C35—H35	119.4
C15—C14—C1	120.0 (2)	C34—C35—H35	119.4
C16—C15—C14	121.20 (19)	C35—C36—C31	120.22 (19)
C16—C15—H15	119.4	C35—C36—H36	119.9
C14—C15—H15	119.4	C31—C36—H36	119.9
			
C31—P—C11—C12	−155.80 (16)	C21—C22—C23—C24	−1.1 (3)
C21—P—C11—C12	92.29 (17)	C22—C23—C24—C25	1.0 (3)
Se—P—C11—C12	−31.70 (17)	C22—C23—C24—C2	−178.22 (19)
C31—P—C11—C16	23.05 (18)	C23—C24—C25—C26	0.1 (3)
C21—P—C11—C16	−88.86 (17)	C2—C24—C25—C26	179.3 (2)
Se—P—C11—C16	147.15 (14)	C24—C25—C26—C21	−1.0 (4)
C16—C11—C12—C13	−0.6 (3)	C22—C21—C26—C25	0.9 (3)
P—C11—C12—C13	178.27 (15)	P—C21—C26—C25	179.49 (18)
C11—C12—C13—C14	−0.6 (3)	C21—P—C31—C36	−164.39 (15)
C12—C13—C14—C15	1.9 (3)	C11—P—C31—C36	83.44 (16)
C12—C13—C14—C1	−176.97 (19)	Se—P—C31—C36	−40.24 (16)
C13—C14—C15—C16	−2.0 (3)	C21—P—C31—C32	17.16 (19)
C1—C14—C15—C16	176.88 (18)	C11—P—C31—C32	−95.02 (17)
C14—C15—C16—C11	0.8 (3)	Se—P—C31—C32	141.31 (15)
C12—C11—C16—C15	0.5 (3)	C36—C31—C32—C33	0.1 (3)
P—C11—C16—C15	−178.35 (15)	P—C31—C32—C33	178.60 (16)
C31—P—C21—C22	−111.10 (18)	C31—C32—C33—C34	−0.9 (3)
C11—P—C21—C22	0.6 (2)	C32—C33—C34—C35	1.0 (3)
Se—P—C21—C22	124.48 (16)	C32—C33—C34—C3	−177.32 (19)
C31—P—C21—C26	70.41 (18)	C33—C34—C35—C36	−0.3 (3)
C11—P—C21—C26	−177.85 (17)	C3—C34—C35—C36	178.00 (19)
Se—P—C21—C26	−54.02 (18)	C34—C35—C36—C31	−0.4 (3)
C26—C21—C22—C23	0.1 (3)	C32—C31—C36—C35	0.5 (3)
P—C21—C22—C23	−178.38 (15)	P—C31—C36—C35	−177.98 (16)

### Hydrogen-bond geometry (Å, °)

**Table:**

*D*—H···*A*	*D*—H	H···*A*	*D*···*A*	*D*—H···*A*
C12—H12···Se	0.95	3.04	3.495 (2)	111
C12—H12···Se^i^	0.95	3.18	3.890 (2)	133
C2—H2B···Se^ii^	0.98	3.09	4.067 (2)	176
C36—H36···Se	0.95	3.13	3.556 (2)	109

Symmetry codes: (i) −*x*+1, −*y*+2, −*z*+1; (ii) *x*+1, *y*, *z*.
